# Receptor Heteromerization Expands the Repertoire of Cannabinoid Signaling in Rodent Neurons

**DOI:** 10.1371/journal.pone.0029239

**Published:** 2012-01-03

**Authors:** Raphael Rozenfeld, Ittai Bushlin, Ivone Gomes, Nikos Tzavaras, Achla Gupta, Susana Neves, Lorenzo Battini, G. Luca Gusella, Alexander Lachmann, Avi Ma'ayan, Robert D. Blitzer, Lakshmi A. Devi

**Affiliations:** 1 Department of Pharmacology and Systems Therapeutics, Mount Sinai School of Medicine, New York, New York, United States of America; 2 Department of Neuroscience and The Friedman Brain Institute, Mount Sinai School of Medicine, New York, New York, United States of America; 3 Systems Biology Center of New York, Mount Sinai School of Medicine, New York, New York, United States of America; 4 Department of Medicine, Mount Sinai School of Medicine, New York, New York, United States of America; Institut National de la Santé et de la Recherche Médicale, France

## Abstract

A fundamental question in G protein coupled receptor biology is how a single ligand acting at a specific receptor is able to induce a range of signaling that results in a variety of physiological responses. We focused on Type 1 cannabinoid receptor (CB_1_R) as a model GPCR involved in a variety of processes spanning from analgesia and euphoria to neuronal development, survival and differentiation. We examined receptor dimerization as a possible mechanism underlying expanded signaling responses by a single ligand and focused on interactions between CB_1_R and delta opioid receptor (DOR). Using co-immunoprecipitation assays as well as analysis of changes in receptor subcellular localization upon co-expression, we show that CB_1_R and DOR form receptor heteromers. We find that heteromerization affects receptor signaling since the potency of the CB_1_R ligand to stimulate G-protein activity is increased in the absence of DOR, suggesting that the decrease in CB_1_R activity in the presence of DOR could, at least in part, be due to heteromerization. We also find that the decrease in activity is associated with enhanced PLC-dependent recruitment of arrestin3 to the CB_1_R-DOR complex, suggesting that interaction with DOR enhances arrestin-mediated CB_1_R desensitization. Additionally, presence of DOR facilitates signaling via a new CB_1_R-mediated anti-apoptotic pathway leading to enhanced neuronal survival. Taken together, these results support a role for CB_1_R-DOR heteromerization in diversification of endocannabinoid signaling and highlight the importance of heteromer-directed signal trafficking in enhancing the repertoire of GPCR signaling.

## Introduction

Cannabinoid receptor signaling is involved in a variety of physiological processes including proliferation and migration, neurite elongation and guidance, synaptogenesis, and cell survival [1]–[4]. The molecular mechanisms that enable a single type of GPCR to achieve such a broad range of functions are of great physiological and clinical relevance, but to date are poorly understood.

CB_1_R is part of the endocannabinoid system that comprises the cannabinoid receptors, their endogenous ligands (the endocannabinoids), the enzymes that produce and inactivate the endocannabinoids, and the endocannabinoid transporters. The two major endocannabinoids, anandamide and 2-arachidonoylglycerol, are lipid-derived messengers generated by the metabolism of arachidonic acid, that acting as retrograde messengers, regulate neuritogenesis and neurite outgrowth [5]. In addition, a recent study reported longer hemopressins as peptide ligands capable of binding to CB_1_R and activating a distinct signal transduction pathway [6]. It is generally accepted that the endocannabinoid system is responsible for shaping the temporal and spatial diversity of cellular responses and hence likely to be involved in adaptive processes and plasticity [1], [5].

CB_1_R belongs to the family A of GPCRs and couples to Gi/o subtypes of heterotrimeric G proteins. CB_1_R activation usually results in the inhibition of adenylyl cyclase activity, inhibition of calcium channels [7], and activation of potassium channels [8]. CB_1_R activation also results in the activation of p42/44 MAP kinase (pERK), downstream of PLCβ [4], [9]. Finally, CB_1_R activation has been shown to lead to recruitment of GPCR kinase 3 and arrestin3, resulting in receptor desensitization [10]. Hence, cannabinoid receptors share a number of common characteristics with opioid receptors, and interactions between these two receptors appear to mutually modulate their activity [11]–[14].

The majority of studies examining interactions between CB_1_R and opioid receptors have focused on the mu opioid receptor (MOR) [15], [16], and relatively few studies have explored the interaction between CB_1_R and DOR. At the cellular level, *in vitro* studies demonstrate cross-desensitization between CB_1_R and DOR at various steps along the signal transduction pathway, including G protein activation and inhibition of adenylyl cyclase activity [17]–[21]. Functional interaction between CB_1_R and DOR has been proposed *in vivo* by studies showing that a DOR antagonist could block the anxiolytic activity of a low dose of the CB_1_R agonist Δ^9^tetrahydrocannabinol (THC) [22] and that mice lacking DOR show a significant increase in CB_1_R activity in several brain regions, as demonstrated by the [^35^S]GTPγS binding assay [23], [24]. These studies support the concept that CB_1_R and DOR interact, and that these interactions impact on CB_1_R activity.

In this study we characterize the direct interaction between CB_1_R and DOR and investigate its consequences on receptor function. We find that CB_1_R and DOR associate form receptor heteromers. Stimulation of CB_1_R within the CB_1_R-DOR heteromer leads to changes in CB_1_R signaling, including recruitment of arrestin3 to the CB_1_R-DOR complex and promotion of an arrestin3-mediated signaling pathway and enhanced neuronal survival. This, in turn, leads to the activation of anti-apoptotic signaling pathways. Taken together, we propose that heteromer-directed signaling leads to the diversification of endocannabinoid signaling by activating distinct signaling pathways with important physiological outcomes such as regulation of cell proliferation and apoptosis.

## Materials and Methods

### Materials

Neuro2A cells endogenously expressing CB_1_R (N2A^CB1R^) were obtained from ATCC. F11 cells were a gift from Dr. D. Felsenfeld (Mount Sinai School of Medicine). Monoclonal anti-phosphoERK, polyclonal anti-ERK, monoclonal anti-myc, polyclonal anti-phosphoDOR(S363), monoclonal anti-phosphoSTAT3 (Ser-727), polyclonal anti-phospho-p90rsk, polyclonal anti-STAT3, polyclonal anti-phosphop70S6K, polyclonal anti-BAD, polyclonal anti-lamin A/C and monoclonal anti-phosphoBAD antibodies were from Cell Signaling Technology Inc. Rabbit anti C-terminal CB_1_R antibody was from Cayman Chemicals. The polyclonal anti-calnexin and anti-FLAG antibodies and pertussis toxin were from Sigma. The anti AP-3δ (anti-delta SA4) monoclonal antibody was from the Developmental Studies Hybridoma Bank, University of Iowa. The monoclonal anti-AP-2α antibody was from BD Biosciences. Rabbit anti C-terminal CB_1_R and goat anti N-terminal CB_1_R polyclonal antibodies were gifts from Dr. Ken Mackie (University of Indiana). The mouse anti-arrestin3 antibody was from Abcam. The rat anti-DOR antibody was generated as described previously [25] and showed no specific signal in ELISA, Western Blot and immunocytochemistry assays with DOR −/− brains (see Figure S1A–C). Monoclonal anti-GAPDH antibody was from Novus Biological. The anti-pericentrin antibody was from Abcam. IRDye 680-labeled anti-rabbit or mouse and IRDye 800-labeled anti-mouse antibodies were from Li-COR. The Alexa 488-conjugated anti-goat, mouse or rabbit, Alexa 594-conjugated anti-rat, goat, mouse or rabbit and Alexa 647-conjugated anti-rabbit antibodies were from Invitrogen. HRP-conjugated anti-rabbit and anti-rat IgG antibodies were from GE Healthcare. Rabbit polyclonal anti-CB_1_R (C-terminal) antibody coupled to agarose beads, rabbit polyclonal anti-myc antibodies and siRNA to arrestin3 were from Santa Cruz Biotechnology. Hu210, U73122, and edelfosine were from Tocris Bioscience. Wild-type mouse DOR and DORΔ15 plasmids were characterized as described previously [26], [27]. The CellTiter-Glo® Luminescent Cell Viability Assay was from Promega. U2OS cells co-expressing prolink/enzyme donor-tagged human DOR and enzyme activator-tagged arrestin3 fusion protein and the PathHunter detection kit were from DiscoveRx.

### Cell Lines and transfections

Neuro2A cells endogenously expressing CB_1_R (N2A^CB1R^) were maintained in DMEM containing 10% FBS and penicillin-streptomycin at 37°C in a humidified 5% CO2 incubator. Neuro2A cells stably expressing either myc-tagged DOR (N2A^CB1R^DOR) or Flag-tagged DORΔ15 (N2A^CB1R^DORΔ15) or transiently expressing the metalloprotease, endothelin converting enzyme-2 (N2A^CB1R^ECE2) were grown in DMEM containing 10% FBS, penicillin-streptomycin and 500 µg/ml G418. F11 cells were grown in F12 media containing 2 mM L-glutamine, 15% FBS, HAT supplement and penicillin-streptomycin. U2OS cells co-expressing ProLink/Enzyme Donor (PK)-tagged human DOR and the Enzyme Activator (EA)-tagged β-arrestin fusion protein (a gift from DiscoveRx), were grown in MEM-alpha (Invitrogen) containing 10% fetal bovine serum, penicillin-streptomycin, 500 µg/ml geneticin and 250 µg/ml hygromycin. Transfections with plasmids and siRNAs were carried out as described [28]. For experiments with primary cortical or striatal neurons, cerebral cortices or striata were dissected from wild-type and DOR lacking (DOR−/−) mice (P1 or P2 mice pups). After trypsin treatment and mechanical trituration, neurons were seeded into poly-L-Lysine coated coverslips (cortical neurons) or 24 well plates (striatal neurons). Cells were grown for 14 days in Neurobasal-B27 media, supplemented with L-glutamine (growth media). On the third day (DIV3) after plating 10 µM cytosine arabinoside was added to inhibit glial growth.

### Generation of shRNA lentiviral vector to DOR

GFP-tagged lentiviral shRNA to murine DOR was generated as described [29] by targeting 364–384 base pairs relative to the start codon of DOR. Infectious particles were produced by transient transfection of 293T cells using Effectene (Qiagen, Carlsbad, CA, USA). After determining the dose at which the virus was able to efficiently infect F11 cells without killing them (as visualized for EGFP fluorescence) we used this dose (5 µl) that also reduces DOR levels by >70% (Rozenfeld and Devi, unpublished) to examine the effect of down-regulation of DOR expression on ERK phosphorylation as described below.

### Animals

DOR +/− embryos (Oprd1^tm1Dgen^; lacking the first exon of DOR [30]) were purchased from the Mutant Mouse Regional Resource Center (MMRRC). DOR −/− mice were generated at the Mount Sinai Mouse Genetics Research Facility by implantation of the embryos into C56/BL6J mice and two successive breeding cycles with wild-type mice from the same background (C56/BL6J). The genotypes of DOR−/− and littermate controls were confirmed by PCR analysis (not shown) and by immunofluorescence, ELISA and Western blotting with the DOR antibody (Figure S1A–C). All protocols complied with the National Institutes of Health Guide for the Care and Use of Laboratory Animals and were approved by the Mount Sinai School of Medicine Institutional Animal Care and Use Committee (Permit # 02-0805). Brain regions from 3 month old male DOR−/− and their wild-type littermates were collected and membranes prepared as described [31]–[33].

### [^35^S]GTPγS binding assays

Assay*s* were carried out essentially as described [31]–[33]. Briefly, membranes from N2A^CB1R^ and N2A^CB1R^DOR cells, and wild type and DOR −/− mice cortices were prepared as described [31]–[33]. The membranes (10 µg) were incubated with increasing concentrations (0–1 µM) of Hu210 in the presence of 100 µM GDP and 0.1 nM [^35^S]GTPγS for one hour at 30°C. Basal binding was determined in the presence of GDP and absence of agonist and cold GTPγS. Non-specific binding was determined by adding 10 µM GTPγS to a parallel set of tubes. Membrane bound radioactivity collected by filtration was detected using a scintillation counter. In experiments examining the effect of the CB_1_R antagonist, SR141716, cells were preincubated with SR141716 (1 µM) for 1 h prior to carrying out the assay.

### Radiolabeled binding assays

Whole cell binding assays were carried out using N2A^CB1R^ and N2A^CB1R^DOR cells as described previously [31]–[33] using [^3^H]SR141716A as a radiolabeled ligand for CB_1_R and [^3^H]DPDPE as a radiolabeled ligand for DOR. CB_1_R levels were 666.3±21.7 fmoles/mg protein in N2A^CB1R^ cells and 637±48 fmoles/mg protein in N2A^CB1R^DOR cells. DOR levels were 1357±28.5 fmoles/mg protein in N2A^CB1R^DOR cells.

### Cell fractionation

N2A^CB1R^ or N2A^CB1R^DOR cells were grown in 6-well plates, serum starved for 6 hours, and stimulated with 100 nM Hu210 for 5 min. Nuclear and cytoplasmic fractions were prepared as described [34]. Immunoblotting for pERK and ERK were performed as described under Western blotting. The purity of the nuclear and cytoplasmic fractions was assessed by immunoblotting for the nuclear protein lamin A/C and the cytoplasmic protein GAPDH as described under Western blotting.

### Coimmunoprecipitation

N2A^CB1R^, N2A^CB1R^DOR or N2A^CB1R^DORΔ15 cells were lysed for 1 h in lysis buffer (1% Triton, 150 mM NaCl, 1 mM EDTA, 1 mM EGTA, and 50 mM Tris-Cl, pH 7.4) containing protease inhibitor cocktail (Sigma). Cell lysates (100–200 µg of protein) were incubated with 1 µg of either rabbit polyclonal anti-CB_1_R (C-terminal) antibody coupled to agarose beads, anti-FLAG or anti-myc antibodies overnight at 4°C. The beads were washed three times with lysis buffer and once with the same buffer without detergent. Proteins were eluted in 60 µL of 2× Laemmli buffer containing 1% 2-mercaptoethanol, resolved by 10% SDS-PAGE, and immunoblotted for the specified antibodies as described under Western blotting.

### Enzyme-linked immunosorbent assay (ELISA)

ELISA to quantify CB_1_R expression in either U2OS cells co-expressing ProLink/Enzyme Donor (PK)-tagged human DOR, Enzyme Activator (EA)-tagged β-arrestin fusion protein and myc-tagged mouse CB_1_R and to quantify DOR expression in wild-type, CB_1_R−/− or DOR−/− cortical membranes was carried out as described [25], [33], [35] using rabbit anti-myc (1∶1000), rat anti-DOR (1∶500), and HRP-conjugated anti-rabbit (1∶2000) or anti-rat antibodies (1∶1000). ELISA to quantify, total CB_1_R levels in N2A^CB1R^, N2A^CB1R^DOR or N2A^CB1R^ECE2 cells was carried out in cells permeabilized with ice-cold methanol for 5 min while cell surface CB_1_R levels were determined in non-permeabilized cells using anti-CB_1_R (1∶500) and HRP-conjugated anti-rabbit (1∶1000) antibodies.

### Confocal microscopy

Confocal microscopy imaging was carried out as described [28]. Primary cortical cells (14DIV) from wild-type or DOR−/− mice, N2A^CB1R^ and N2A^CB1R^DOR cells transfected without or with a plasma membrane marker (a GFP fused with a prenylation site; Mb-GFP) or overexpressing arrestin3-eGFP (and treated without or with 100 nM Hu210 for 5 min) or F11 cells infected with scramble or DOR shRNA lentivirus were fixed with either 4% paraformaldehyde (PFA) in phosphate buffered saline (PBS) or with methanol and permeabilized with 0.1% Triton-X-100. The following antibodies were used as primary antibodies as described in the figure legends: goat anti-N-terminal CB_1_R (1∶500), rabbit anti-C-terminal CB_1_R (1∶500), rat anti-DOR (1∶500), mouse anti-myc (1∶1000), rabbit anti-phospho ERK (1∶1000), mouse anti-pericentrin (1∶1000), mouse anti-AP-3δ (1∶1000), mouse anti-AP-2α (1∶1000). The following antibodies were used as secondary antibodies: Alexa 488-conjugated anti-goat, rabbit or mouse (1∶1000), Alexa 594-conjugated anti-rat, mouse, goat or rabbit (1∶1000), Alexa 647-conjugated anti- rabbit (1∶1000). Slides were visualized with a Leica TCS SP5 confocal microscope. Images were acquired with an ×63/1.32 PL APO objective lens, and analyzed in sequential scanning mode. For studies examining colocalization of CB_1_R with the membrane marker Mb-GFP, colocalization was examined in horizontal and vertical sections of the cells. Metamorph software (Molecular Devices) was used for quantification of colocalization in multiple horizontal sections of 8 individual cells/group. The percentage of CB_1_R pixels colocalized with Mb-GFP was calculated in each section and the average for each cell represented in a graph (Figure S2C).

### Phospho-ERK assays

Phospho-ERK assays were carried out as described in [28], [36]. Briefly, N2A^CB1R^, N2A^CB1R^DOR or N2A^CB1R^DORΔ15 cells alone or transiently transfected with control or arrestin3-targeting siRNA, or F11 cells infected with scramble or DOR shRNA lentivirus (∼40,000 cells/well) were seeded on 24-well plates. The next day, cells were starved for at least 4–6 h in serum-free medium prior to stimulation with Hu210 for the indicated times at the indicated concentrations. In some cases cells were preincubated for 30 min with indicated kinase inhibitors, followed by treatment with Hu210 in the presence of these inhibitors. In experiments examining the effect of pertussis toxin (PTX) pretreatment, cells were preincubated for 16 h with PTX (15 ng/ml) prior to carrying out the phospho-ERK assay. Cells were solubilized by directly adding 1× SDS buffer pre-warmed to 65°C, followed by sonication with a microtip for 5 sec and subjected to Western Blotting as described below.

### Western blotting analyses

Western blots were carried out as described in [28], [36]. Briefly, proteins in cell lysates (30 µg protein; Laemmli buffer containing 1% 2-mercaptoethanol) were resolved in 10% SDS-PAGE gels and subjected to Western blot analysis. The following antibodies were used as primary antibodies: rabbit anti-phosphoERK (1∶1000), mouse anti-ERK (1∶1000), rabbit anti-CB_1_R (C-terminal; 1∶500), mouse anti-arrestin3 (1∶500), rabbit anti-phosphoDOR Ser 363 (1∶1000), mouse anti-myc (1∶1000), rabbit anti-lamin A/C (1∶2000), mouse anti-GAPDH (1∶2000), rabbit anti-STAT3 (1∶1000), mouse anti-phosphoSTAT3 (1∶1000), rabbit anti-phospho p70S6K (1∶1000), rabbit anti-phospho-p90rsk (1∶1000), rabbit anti-phosphoBAD (1∶1000), mouse anti-BAD (1∶1000), rabbit anti-calnexin (1∶1000). In experiments examining the specificity of rat anti-DOR antibodies, cortical membranes from wild-type and DOR−/− mice (30 µg protein) were solubilized in Laemmli buffer containing 1% 2-mercaptoethanol, followed by sonication with a microtip for 5 sec and subjected to Western Blotting using rat anti-DOR (1∶1000) antibodies. The following antibodies were used as secondary antibodies: IRDye 680-labeled anti-rabbit (1∶10,000), IRDye 800-labeled anti-mouse (1∶10,000) and IRDye 680-labeled anti-rat (1∶10,000). Blotting, imaging and band intensity measurements were performed using the Odyssey imaging system (LI-COR, Lincoln, NE) according to the manufacturer's protocols.

### Arrestin recruitment assay

U2OS cells co-expressing ProLink/Enzyme Donor (PK)-tagged human DOR and the Enzyme Activator (EA)-tagged β-arrestin fusion protein, were transfected with myc-tagged mouse CB_1_R using FuGene as per manufacturer's protocol (Roche Diagnostics). Expression of myc-CB_1_R was determined by ELISA as described above. The day after transfection, cells were seeded in 96 well plates (20,000/well) in growth media. The following day media was removed and replaced with 90 µl growth media without antibiotics. Hu210 (0–10 µM in 10 µl) was added to the wells and incubated for 30 min. β-arrestin recruitment was determined using the PathHunter Detection Kit, as described in the manufacturer's protocol. Samples were read on a luminescence plate reader (Perkin-Elmer).

### Survival and caspase-3 assays

N2A^CB1R^ and N2A^CB1R^DOR cells (20,000 cells/well) were plated in complete media (+FBS) on poly-L-lysine coated 24-well plates. Next day, cells were placed in starving media (media containing 0.1% FBS). The cells were treated with 1 µM Hu210. Control cells were not subjected to any drug treatment. Fresh ligands were added daily without changing the medium. The number of live cells was determined by the Trypan Blue exclusion method. For the caspase-3 assay, N2A^CB1R^ and N2A^CB1R^DOR cells (1×10^6^ cells/well) were plated in complete media on 6-well plates. Next day, cells were placed in starving media and treated with 1 µM Hu210. Control cells were not subjected to any drug treatment. Fresh ligands were added daily without changing the medium. After 3 and 8 days, the medium was removed, wells washed with PBS, cells collected and assayed for caspase-3 activity using a colorimetric CaspACE TM assay system (Promega Corporation, Madison,WI) as per manufacturer's protocol.

For experiments involving the survival of striatal neurons, the CB_1_R receptor antagonist, AM251 (10 µM) was added to the growth media at DIV7 and cellular viability was assessed at DIV10 by monitoring intracellular ATP using the CellTiter-Glo® Luminescent Cell Viability Assay as described in the manufacturer's protocol.

### Data Analysis

Data were analyzed (and EC_50_ and E_max_ values were determined) using Prism 4.0 (Graph Pad, San Diego, CA, U.S.A.). Student's t-test was used to determine statistical significance.

## Results and Discussion

### Increased CB_1_R signaling in the absence of DOR

**Figure 1:**
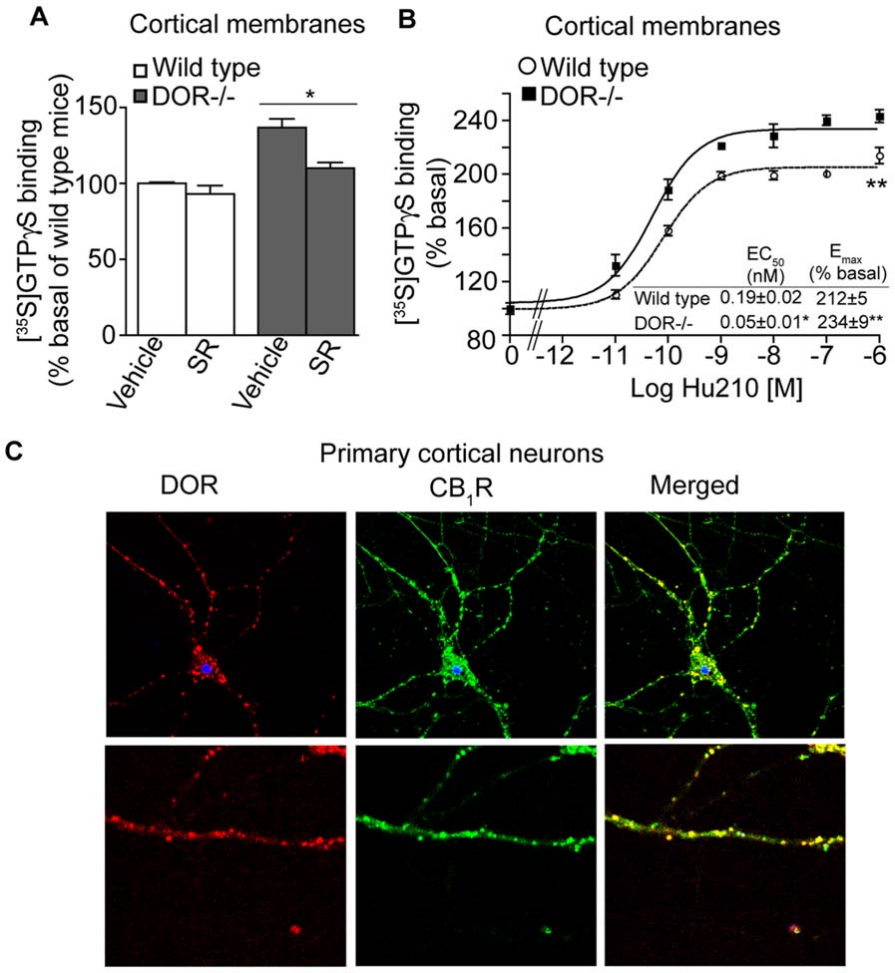
Increased CB_1_R activity in the absence of DOR. **A**, Basal [^35^S]GTPγS binding was measured in cortical membranes from wild-type and DOR −/− mice. Membranes from cortices were prepared as described [31]–[33], treated with vehicle or 1 µM SR141716 (SR) for 1 hour and subjected to [^35^S]GTPγS binding as described in Methods. Basal [^35^S]GTPγS binding/10 µg protein in vehicle treated membranes is taken as 100%. Data represent Mean ± SEM (n = 3 individual animals in triplicate). Statistically significant differences between vehicle and SR141716 treatment are indicated *p<0.05, (t test). **B**, [^35^S]GTPγS binding assay in cortical membranes from wild-type and DOR −/− mice. Membranes were treated with increasing concentrations of the CB_1_R agonist Hu210 (0–1 µM) and [^35^S]GTPγS binding was measured as described in Methods. EC_50_ and E_max_ values were calculated using GraphPad Prism software. Data represent Mean ± SEM (n = 3 individual animals in triplicate). *p<0.05; **p<0.01 for DOR−/− vs wild-type (t test). **C**, Localization of endogenous CB_1_R and DOR in mouse primary cortical cells, 14DIV. Cells fixed with 4%PFA in PBS and permeablized with 0.1% Triton, were immunostained with the goat polyclonal anti-CB_1_R(N-terminal) antibody (1∶500; green) and rat polyclonal anti-DOR antibody (1∶500; red) and visualized using Alexa 488-conjugated anti-goat (1∶1000) and Alexa 594-conjugated anti-rat (1∶1000) secondary antibodies using confocal microscopy as described in Methods. Representative figure from 3 independent experiments shown.

In order to examine the extent to which DOR would affect CB_1_R signaling, we compared classic G protein-mediated signaling in cortical membranes from wild type mice to signaling in membranes from mice lacking DOR (DOR−/−), using the [^35^S]GTPγS binding assay. We found that in the absence of DOR there was a small but significant increase in basal [^35^S]GTPγS binding; this increase could be partially blocked by treatment with the CB_1_R inverse agonist SR141716 (Fig. 1A). Absence of DOR also led to a ∼3-fold increase in the potency of Hu210 (a CB_1_R agonist) to induce [^35^S]GTPγS binding (Fig. 1B). These findings suggest that the presence of DOR regulates CB_1_R-mediated G-protein activation. This could be due to a functional interaction between CB_1_R and DOR arising either from an indirect intercellular regulatory mechanism (with the two receptors interacting within the same neuronal circuit) or from direct intracellular cross-talk (such as altered G protein coupling and/or physical interaction).

### CB_1_R and DOR co-localize in primary cortical neurons

Since the presence of intracellular cross-talk would require co-expression of these two receptors, we examined expression and localization of CB_1_R and DOR in primary cortical neurons. Using an antibody that selectively recognizes DOR (as evident from confocal microscopy analysis, ELISA and Western blotting assays either with cells expressing one or both receptors or membranes from DOR−/− mice and their littermate controls; Figure S1A–C) we found that DOR is expressed in primary cortical neurons (that also expresses CB_1_R) and that there is a large degree of co-localization between CB_1_R and DOR within neuronal cell bodies and in the processes (Fig. 1C). These findings suggest the possibility of intracellular interactions between CB_1_R and DOR in cells that express both receptors.

### CB_1_R and DOR form interacting complexes

**Figure 2:**
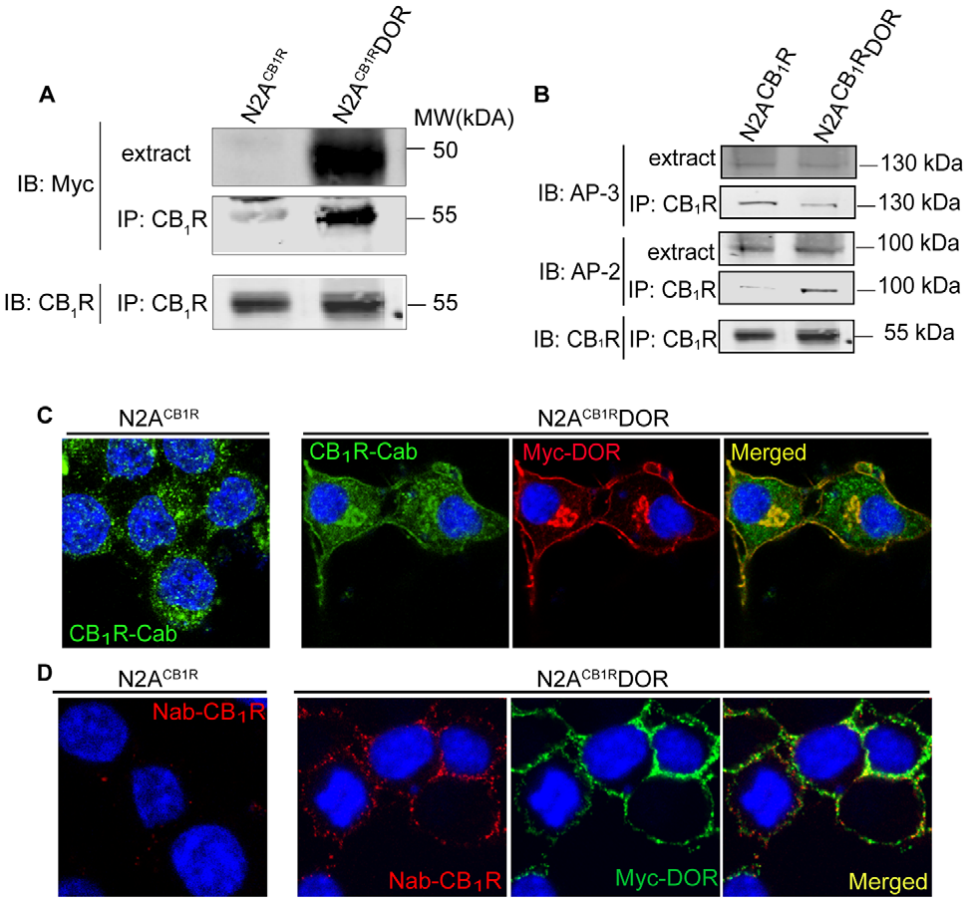
Association between CB_1_R and DOR alters CB_1_R localization. **A**, Lysates (100–200 µg) from N2A^CB1R^ and N2A^CB1R^DOR cells were subjected to immunoprecipitation with 1 µg of anti-CB_1_R (C-terminal) antibody, the immunoprecipitates were resolved on 10% SDS-PAGE and probed for the presence of myc-DOR using mouse monoclonal anti-myc antibody (1∶1000) and for CB_1_R using rabbit polyclonal anti-CB_1_R (C-terminal) antibody (1∶500) as described in Methods. IRDye 680 anti-rabbit and IRDye 800 anti-mouse were used as secondary antibodies (1∶10,000). Representative of 3 independent experiments shown. **B**, CB_1_R-DOR complexes exhibit greater interaction with AP-2 than AP-3. Lysates (100–200 µg) from N2A^CB1R^ and N2A^CB1R^DOR cells were subjected to immunoprecipitation using 1 µg of an anti-CB_1_R (C-terminal) antibody as described in Methods. The immunoprecipitates were resolved on 10% SDS-PAGE and probed for the presence of AP-3 (1∶1000), AP-2 (1∶1000) and CB_1_R (C-term) (1∶500) using specific antibodies as described in Methods. IRDye 680 anti-rabbit and IRDye 800 anti-mouse were used as secondary antibodies (1∶10,000). Representative of 3 independent experiments shown. **C**, Localization of endogenous CB_1_R in N2A^CB1R^ and of CB_1_R and DOR in N2A^CB1R^DOR cells. Cells fixed with 4%PFA in PBS and permeablized with 0.1% Triton, were stained with the rabbit polyclonal anti-CB_1_R (C-terminal) antibody (1∶500; green) and the mouse monoclonal anti-myc antibody (1∶1000; red) and visualized using Alexa 488-coupled anti-rabbit or Alexa 594-coupled anti-mouse secondary antibodies (1∶1000) using confocal microscopy as described in Methods. Representative of 3 independent experiments shown. **D**, Cell surface staining of endogenous CB_1_R and stably expressed DOR in N2A^CB1R^DOR cells. N2A^CB1R^ and N2A^CB1R^DOR cells were stained with a goat polyclonal anti-CB1R (N-terminal) antibody (1∶500) and mouse monoclonal anti-myc antibodies (1∶1000) prior to fixation of the cells to label cell surface receptors, as described [28]. After fixation, cells were visualized with Alexa 594-coupled anti-goat and Alexa 488-coupled anti-mouse secondary antibodies (1∶1,000) using confocal microscopy as described in Methods. Representative of 3 independent experiments shown.

Next, we examined if CB_1_R and DOR exist in an interacting complex using co-immunoprecipitation analysis of membranes from Neuro2A cells that endogenously express CB_1_R (N2A^CB1R^) and from Neuro2A cells stably expressing myc-DOR (N2A^CB1R^DOR). We found that myc-DOR could be detected in CB_1_R immunoprecipitates from N2A^CB1R^DOR cells but not from N2A^CB1R^ cells (Fig. 2A). Conversely, CB_1_R could be detected in myc-DOR immunoprecipitates from N2A^CB1R^DOR cells but not from N2A^CB1R^ cells (Figure S1D). These results support a direct interaction between DOR and CB_1_R and are in agreement with our previous study using a bioluminescence resonance energy transfer (BRET) assay that showed that CB_1_R and DOR exist in close proximity for interaction in live cells [15]. In a previous study we had reported that CB_1_R, when expressed alone, associates with the adaptor protein AP-3 (involved in sorting to the endolysosomes) [28]. Here we find that the level of AP-3 associated with CB_1_R-DOR is significantly lower as compared to CB_1_R (Fig. 2B). In contrast, we find that the level of the adaptor protein AP-2 (involved in GPCR endocytosis [37]) associated with CB_1_R-DOR is substantially higher as compared to CB_1_R (Fig. 2B). These results, showing that the association of DOR with CB_1_R results in differential recruitment of adaptor proteins, suggest that CB_1_R-DOR interactions would lead to altered localization of CB_1_R.

### Association with DOR affects CB_1_R localization

Given the differential recruitment of adaptor proteins by CB_1_R-DOR, and the finding that when expressed alone, CB_1_R is mostly present in intracellular vesicles [37], [38], whereas DOR is found primarily at the cell surface, we examined changes in the subcellular localization of CB_1_R in the presence of DOR. Consistent with previous findings, we observed that in N2A^CB1R^ cells, endogenous CB_1_R is not detected at the plasma membrane, but is mostly localized in intracellular compartments (Fig. 2C, left). In contrast, in N2A^CB1R^DOR cells, endogenous CB_1_R is localized at the plasma membrane (Fig. 2C, right). This DOR-induced plasma membrane localization of CB_1_R was confirmed by incubating non-permeabilized living N2A^CB1R^ and N2A^CB1R^DOR cells with primary antibodies raised against extracellular epitopes of CB_1_R and myc-DOR (Fig. 2D). Under these conditions, there was virtually no CB_1_R staining in N2A^CB1R^ cells (Fig. 2D, left) whereas both CB_1_R and DOR were readily detected at the plasma membrane of N2A^CB1R^DOR cells (Fig. 2D, right).

We also quantified CB_1_R levels at the plasma membrane in N2A^CB1R^ and N2A^CB1R^DOR cells by measuring the extent of colocalization with a plasma membrane marker, a GFP fused with a prenylation site (Mb-GFP) (Figure S2A–C). We found <5% of CB_1_R colocalized with Mb-GFP in N2A^CB1R^ cells, whereas >40% of CB_1_R staining colocalized with the plasma membrane marker in N2A^CB1R^DOR cells. In order to determine the specificity of the change in CB_1_R localization, we measured the changes in cell surface CB_1_R immunoreactivity in Neuro2A cells stably expressing the metalloprotease ECE2, a type II transmembrane protein that is transported to the plasma membrane via the secretory pathway (Gagnidze and Devi, unpublished). While the presence of DOR led to a significant increase in cell surface CB_1_R immunoreactivity, the presence of ECE2 did not affect CB_1_R plasma membrane levels (Figure S2D), confirming the specificity of CB_1_R-DOR interaction in modulating CB_1_R cell surface expression. We also examined the effect of decreasing endogenous DOR expression (by shRNA-expressing lentivirus) in F11 cells (that express both DOR [39] and CB_1_R [40]) on the localization of endogenous CB_1_R. We found that in cells transduced with a DOR shRNA-expressing lentivirus, there was no detectable CB_1_R at the cell surface (Figure S2E) indicating that expression of DOR modulates the localization of CB_1_R. These results are consistent with the notion that DOR associates with CB_1_R and facilitates its cell surface localization.

### Co-expression of CB_1_R and DOR affects CB_1_R-mediated ERK signaling

**Figure 3:**
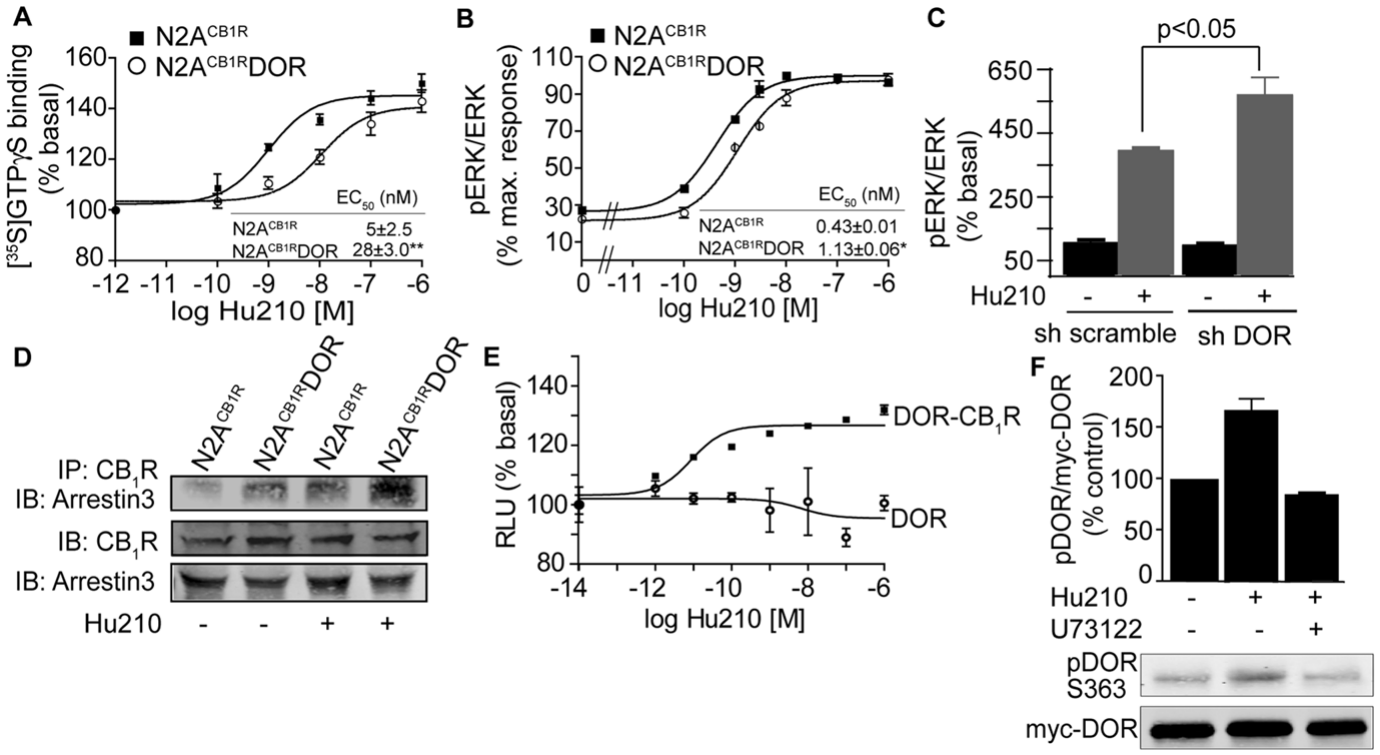
PLC-dependent arrestin3 association with CB1R-DOR complex. **A**, [^35^S]GTPγS binding assay in membranes from N2A^CB1R^ and N2A^CB1R^DOR cells. Membranes (10 µg) were treated with indicated concentrations of the CB_1_R agonist Hu210. [^35^S]GTPγS binding was measured as described in Methods. EC_50_ and E_max_ values were calculated using GraphPad Prism software. Data represent Mean ± SEM (n = 3 independent experiments in triplicate). **B**, Dose-response of Hu210-mediated ERK phosphorylation in N2A^CB1R^ and N2A^CB1R^DOR cells. Starved N2A^CB1R^ and N2A^CB1R^DOR cells seeded in 24 well-plates were treated with indicated concentrations of Hu210 for 5 minutes. Cell lysates (30 µg protein) were analyzed by Western blotting and probed for the levels of pERK (1∶1000) and ERK (1∶1000) as described in Methods. IRDye 680 anti-rabbit and IRDye 800 anti-mouse were used as secondary antibodies (1∶10,000). EC_50_ and E_max_ values were calculated using GraphPad Prism software. Data represent Mean ± SEM (n = 3 independent experiments). *p<0.05 for N2A^CB1R^DOR vs N2A^CB1R^ (t test). **C**, Effect of DOR down-regulation on ERK phosphorylation. F11 cells transduced with the DOR shRNA expressing lentivirus were starved for 4–6 h and treated with Hu210 (100 nM) for 5 min. Cell lysates (30 µg protein) were analyzed by Western blotting and probed for the levels of pERK (1∶1000) and ERK (1∶1000) as described in Methods. IRDye 680 anti-rabbit and IRDye 800 anti-mouse were used as secondary antibodies (1∶10,000). Data from 3 independent experiments is shown. *p<0.05 (t test). **D**, Examination of arrestin3 interaction with CB_1_R after Hu210 treatment. N2A^CB1R^ and N2A^CB1R^DOR, starved for 4 hours were stimulated with 100 nM Hu210 for 5 minutes and cell lysates prepared as described in Methods. Lysates (30 µg protein) were subjected to either Western blotting using rabbit anti-CB1R (C-terminal 1∶500) and mouse anti-arrestin 3 antibodies (1∶500) or to immunoprecipitation using 1 µg of agarose-coupled anti-CB_1_R (C-terminal) antibody. Immunoprecipitates were probed for arrestin3 levels by Western blot using the mouse anti-arrestin 3 antibody. IRDye 680 anti-rabbit and IRDye 800 anti-mouse were used as secondary antibodies (1∶10,000). Representative of 3 independent experiments shown. **E**, Effect of Hu210 on arrestin recruitment. U2OS cells co-expressing ProLink/Enzyme Donor (PK)-tagged DOR and the Enzyme Activator (EA)-tagged arrestin3 fusion protein without or with CB_1_R were treated with indicated concentrations of Hu-210. Arrestin3 recruitment was determined using the PathHunter Detection Kit as described in Methods. Data represent Mean ± SEM (n = 4). **F**, Effect of PLC inhibitor (U73122) on DOR phosphorylation at serine 363 after Hu210 treatment. N2A^CB1R^DOR cells were starved for 4–6 hours, and incubated with vehicle (DMSO) or U73122 (1 µM) for 30 minutes, then stimulated with 100 nM Hu210 for 5 minutes. Cell lysates (30 µg protein) were subjected to Western blotting using rabbit polyclonal phosphoDOR Ser 363 (1∶1000), mouse monoclonal anti-myc (1∶1000) antibodies and IR Dye 680 anti-rabbit and IR Dye 800 anti-mouse secondary antibodies (1∶10,000) as described in Methods. Data represent Mean ± SEM (n = 3).

We examined if the association with DOR altered CB_1_R signaling by examining CB_1_R-mediated G protein coupling in N2A^CB1R^DOR cells. The presence of DOR led to a ∼10-fold decrease in the potency of Hu210 (a CB_1_R agonist) to induce [^35^S]GTPγS binding (Fig. 3A). We also examined the presence of DOR on CB_1_R-mediated modulation of pERK levels; pERK is a known downstream effector of CB_1_R. We find that the presence of DOR led to a 3-fold decrease in the potency of Hu210 to phosphorylate ERK (Fig. 3B & Figure S3A). In order to confirm that CB_1_R signal modulation was solely due to DOR co-expression, we downregulated DOR using shRNA expressing lentivirus in F11 cells that endogenously co-express CB_1_R and DOR and we examined pERK levels in response to CB_1_R activation. We find that downregulation of DOR leads to enhancement in pERK levels as compared to scrambled shRNA treatment (Fig. 3C & Figure S3B). Taken together these results are consistent with the idea that association with DOR leads to reduction in CB_1_R signaling; this could be due to recruitment of distinct sets of signaling molecules.

### CB_1_R activation leads to arrestin3 recruitment in cells co-expressing CB_1_R and DOR

We hypothesized that reduced CB_1_R signaling in N2A^CB1R^DOR cells could be due to altered arrestin3 recruitment to CB_1_R, as previous studies had demonstrated arrestin recruitment to opioid receptor heteromers [36]. We tested this by co-immunoprecipitation and found that the levels of arrestin3 associated with CB_1_R in the presence of DOR are higher than in the absence of DOR (Fig. 3D). Interestingly, activation of CB_1_R leads to a further increase (>2-fold higher in N2A^CB1R^DOR cells as compared to N2A^CB1R^ cells) in arrestin3 recruitment (Fig. 3D). Further support for this comes from studies using the PathHunter arrestin assay (DiscoveRx). In this assay, DOR is fused to one fragment of β-galactosidase (β-gal), and arrestin3 is fused to its complementary fragment. Upon arrestin recruitment to DOR the complementation of the two β-gal fragments result in the restoration of enzyme activity. When CB_1_R (untagged) is expressed in these cells, we find that Hu210 treatment leads to a dose-dependent increase in β-gal activity; this is seen only in cells co-expressing CB_1_R and DOR and not in cells only expressing DOR (Fig. 3E) confirming activity dependent recruitment of arrestin3 to the heteromer. We directly examined enhanced arrestin3 recruitment to the CB_1_R-DOR heteromer by confocal microscopy analysis of N2A^CB1R^DOR cells expressing arrestin3-eGFP. We found that Hu210 treatment induces arrestin3 translocation to the cell membrane to a greater extent in N2A^CB1R^DOR cells as compared to N2A^CB1R^ alone (Figure S2F). These findings suggest that the presence of DOR enhances arrestin3 binding to CB_1_R in cells co-expressing CB_1_R and DOR.

Next, we tested if DOR phosphorylation at Ser363 is differentially affected by CB_1_R-DOR interactions since this site is reported to be involved in desensitization and recruitment of arrestin3 [41]. We find that in N2A^CB1R^DOR cells, CB_1_R stimulation induced an increase in DOR Ser363 phosphorylation (Fig. 3F). Interestingly, PLC inhibitors, U73122 or edelfosine, completely block this effect (Fig. 3F & Figure S3C). These results suggest that the presence of DOR in N2A^CB1R^DOR cells leads to phosphorylation of DOR C-tail resulting in arrestin3 recruitment to the CB_1_R-DOR complex and PLC activity appears to be involved in this process.

### Promotion of arrestin3-dependent signaling in cells co-expressing CB_1_R and DOR

**Figure 4:**
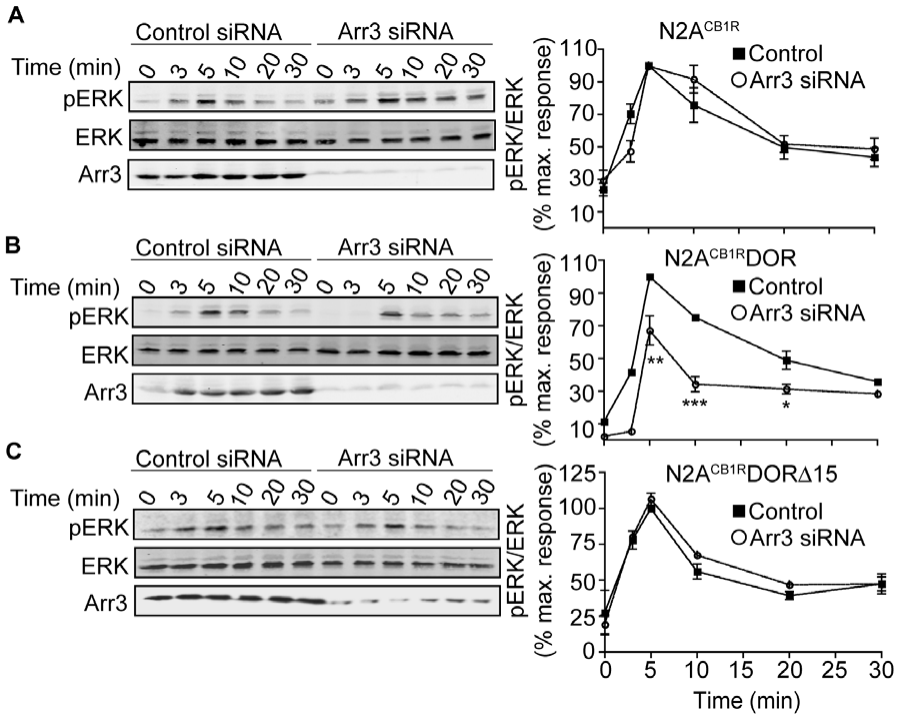
Engagement of arrestin3-dependent signaling in N2A^CB1R^DOR. Time course of Hu210-mediated ERK phosphorylation in **A**, N2A^CB1R^; **B**, N2A^CB1R^DOR; and **C**, N2A^CB1R^DORΔ15 cells transfected with control or arrestin3-targeting siRNA. N2A^CB1R^ alone or stably expressing either DOR or DORΔ15, transfected with a control or arrestin3-targeting siRNA, were starved for 4 hours, then stimulated with 100 nM Hu210 for the indicated times. Cell lysates (30 µg protein) were subjected to Western blotting for the levels of ERK (1∶1000), pERK (1∶1000), and arrestin3 (1∶500) as described in Methods. IRDye 680 anti-rabbit and IRDye 800 anti-mouse were used as secondary antibodies (1∶10,000). Data represent Mean ± SEM (n = 3); *p<0.05; **p<0.01; ***p<0.001, for control vs Arr3 siRNA (t test).

Next we examined arrestin3-dependent signaling by focusing on MAPK phosphorylation since previous studies have reported arrestin3 to mediate G protein-independent ERK activation [36], [42]. Interestingly, arrestin3 downregulation by RNAi led to a significant decrease in pERK levels in N2A^CB1R^DOR, but failed to do so in N2A^CB1R^ cells (Fig. 4A & 4B) underscoring a role for arrestin3 in ERK signaling. We next examined the contribution of DOR C-tail to arrestin3-mediated signaling. We find that in N2A^CB1R^DORΔ15 cells (in which the C-term tail of DOR is deleted, but still forms heteromers with CB_1_R, see Figure S1D), arrestin3 down-regulation did not affect Hu210-mediated ERK phosphorylation (Fig. 4C). Since these 15 amino acids in the C-terminus contain residues important for receptor phosphorylation and downregulation [26], these results indicate that the C-terminal tail of DOR is required for recruiting and facilitating arrestin-dependent signaling by the heteromer. Taken with our previous observations that CB_1_R activation leads to phosphorylation of DOR at the C-terminal tail Ser363 (Fig. 3F), these results elucidate a possible mechanism for CB_1_R-DOR heteromer-mediated signaling that involves recruitment of novel, heteromer-specific signalosomes by DOR C-tail. Interestingly, we find that Hu210-mediated ERK phosphorylation is blocked by pretreatment with pertussis toxin in both in N2A^CB1R^ and in N2A^CB1R^DOR cells (Figure S3D). This suggests that, in these cells, Hu210 treatment leads to arrestin3 mediated G-protein dependent ERK activation. The finding that cannabinoid stimulation leads to DOR phosphorylation-dependent arrestin3 recruitment indicates that direct interaction with DOR contributes to the desensitization of CB_1_R. In addition, in contrast to a single PLCβ-mediated signaling pathway, CB_1_R in the presence of DOR engages both PLC- and arrestin3-mediated pathways to phosphorylate ERK.

### Novel signaling pathways activated in cells co-expressing CB_1_R and DOR

**Figure 5:**
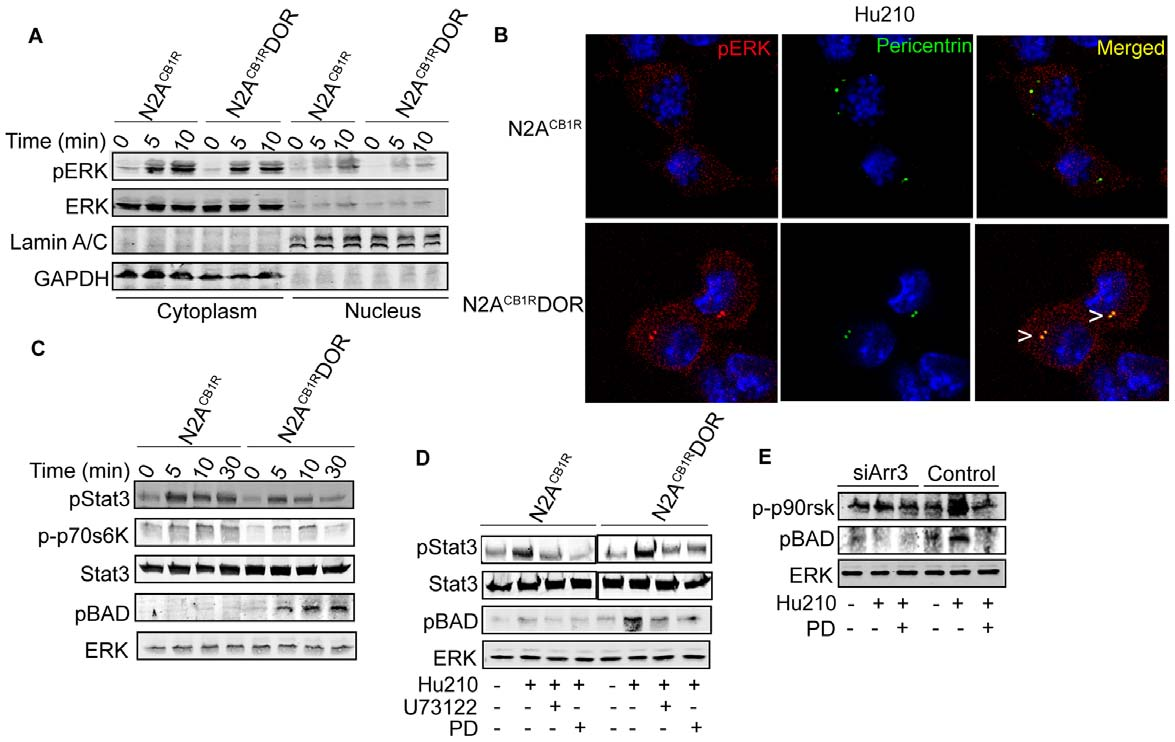
Role of arrestin3 and ERK substrates in cannabinoid signaling by the CB_1_R-DOR heteromer. **A–B**, Effect of CB1R-DOR heteromerization on subcellular localization of pERK. **A**, N2A^CB1R^ and N2A^CB1R^DOR cells were treated with Hu210 (100 nM; 0, 5 or 10 min), cytoplasmic and nuclear extracts were prepared as described in Methods and analyzed (30 µg protein) by Western blotting with pERK (1∶1000), ERK (1∶1000), lamin A/C (1∶2000), and GAPDH (1∶2000) antibodies. IRDye 680 anti-rabbit and IRDye 800 anti-mouse were used as secondary antibodies (1∶10,000). **B**, N2A^CB1R^ and N2A^CB1R^DOR cells treated with Hu210 (100 nM; 5 min) were immunostained with pERK (1∶1000, red) and pericentrin (1∶1000, green) antibodies and visualized using Alexa 488-conjugated anti-rabbit (1∶1000) and Alexa 594-conjugated anti-mouse (1∶1000) secondary antibodies using confocal microscopy as described in Methods. Representative of 3 independent experiments shown. **C**, Time course of phosphorylation of pERK substrates. Lysates (30 µg protein) from N2A^CB1R^ and N2A^CB1R^DOR cells treated with Hu210 (100 nM; 0, 5, 10 or 30 min) were analyzed by Western blotting with STAT3 (1∶1000), phosphoSTAT3 (1∶1000), phospho-p70S6K (1∶1000), phospho-p90rsk (1∶1000) and phosphoBAD (1∶1000) antibodies. IRDye 680 anti-rabbit and IRDye 800 anti-mouse were used as secondary antibodies (1∶10,000). ERK (1∶1000) is used as a loading control. Representative of 3 independent experiments shown. **D**, Involvement of PLC and MEK in the phosphorylation of STAT3 and BAD. Lysates (30 µg protein) from N2A^CB1R^ and N2A^CB1R^DOR cells treated with 100 nM Hu210 for 5 min in the absence or presence of U73122 (1 µM) or PD98059 (PD, 10 µM) were analyzed by Western blotting with STAT3 (1∶1000), phosphoSTAT3 (1∶1000), and phosphoBAD (1∶1000) antibodies. IRDye 680 anti-rabbit and IRDye 800 anti-mouse were used as secondary antibodies (1∶10,000). ERK (1∶1000) is used as a loading control. Representative of 3 independent experiments shown. **E**, Involvement of arrestin3 in BAD phosphorylation. Arrestin3 was down-regulated in N2A^CB1R^DOR cells by transfection with a siRNA. These cells were stimulated with Hu210 (100 nM) for 5 min, in the absence or presence of PD (10 µM). Lysates (30 µg protein) were analyzed by Western blotting with phospho-p90rsk (1∶1000) and phosphoBAD (1∶1000) antibodies. IRDye 680 anti-rabbit and IRDye 800 anti-mouse were used as secondary antibodies (1∶10,000). ERK (1∶1000) is used as a loading control. Representative of 3 independent experiments shown.

**Figure 6:**
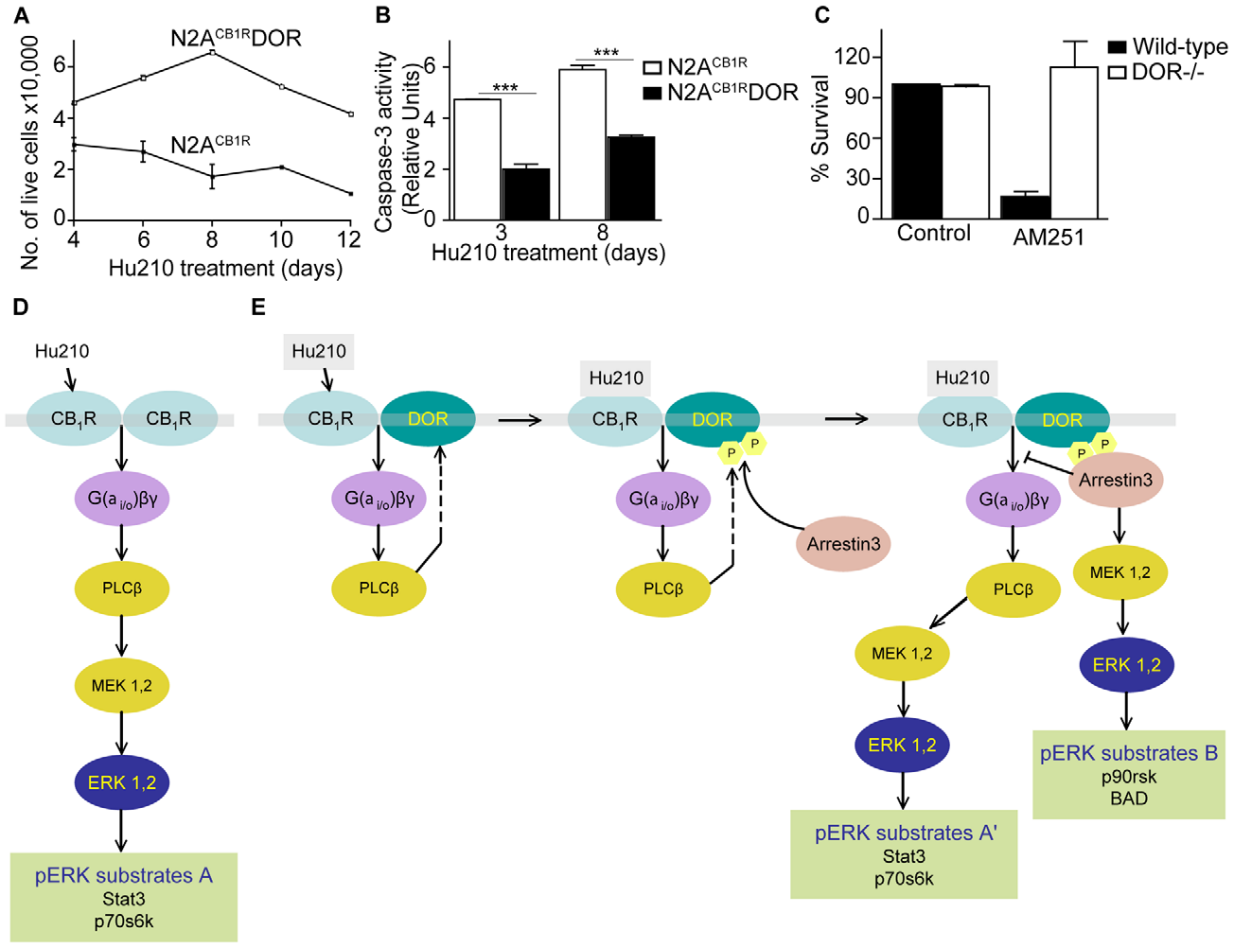
CB_1_R-DOR heteromerization promotes cell survival. **A**, Hu210-treated N2A^CB1R^DOR cells exhibit increased survival compared to N2A^CB1R^ cells. N2A^CB1R^ or N2A^CB1R^DOR cells were treated with 1 µM Hu210 for the indicated days and survival measured by trypan blue exclusion as described in Methods. Data represent Mean ± SEM (n = 4 in triplicate). **B**, Hu210-treated N2A^CB1R^DOR cells exhibit lower apoptosis as compared to N2A^CB1R^ cells. Apoptosis of N2A^CB1R^ or N2A^CB1R^DOR treated for 3 or 8 days with 1 µM Hu210 was measured using caspase-3 activity assay as described in Methods. Data represent Mean ± SEM (n = 4 in triplicate); ***p<0.001 N2A^CB1R^DOR vs N2A^CB1R^ (t test). **C**, CB_1_R antagonist treatment decreases neuronal survival of striatal neurons from wild-type but not DOR−/− mice. Primary striatal neurons from wild-type or from DOR−/− mice were prepared as described in Methods. The CB_1_R antagonist AM251 (10 µM) was added to the growth media at DIV7 and cellular viability assessed at DIV10 as described in Methods. Data represent Mean ± SEM (n = 2–4) **D–E**, A schematic of the signaling pathways emanating from CB_1_R in N2A^CB1R^ (**D**) and N2A^CB1R^DOR (**E**) cells. Activation of CB_1_R in N2A^CB1R^DOR cells leads to differential activation of signaling molecules and phosphorylation of ERK substrates.

We have previously shown that GPCR heteromerization can lead to changes in the localization of pERK [36]. We examined the location of pERK in N2A^CB1R^DOR and N2A^CB1R^ cells following CB_1_R activation and found a small but consistent increase in pERK in the nucleus of N2A^CB1R^ but not in N2A^CB1R^ DOR cells (Fig. 5A). However, upon further examination of N2A^CB1R^DOR cells, it was clear that pERK exhibited a distinct punctate distribution in a juxtanuclear compartment and this was not seen in N2A^CB1R^ cells (Fig. 5B). This pERK enriched compartment in N2A^CB1R^DOR cells was identified as centrosomes using the centrosome marker, pericentrin (Fig. 5B). This is interesting since previous studies have reported the presence of arrestin3 and associated signaling molecules in centrosomes [43], [44]. This, taken with the reports that arrestin3 can regulate the phosphorylation of BAD and thereby activate anti-apoptotic signaling pathways [45] led us to predict that pERK substrates involved in cell survival would be differentially phosphorylated in N2A^CB1R^DOR vs. N2A^CB1R^ cells. To test this we examined the phosphorylation levels of BAD, STAT3 and p70S6K as potential pERK substrates [4], [9]. We found that CB_1_R activation leads to substantially enhanced phosphorylation of BAD and reduced phosphorylation of STAT3 and p70S6K in N2A^CB1R^DOR cells as compared N2A^CB1R^ cells (Fig. 5C & D). Furthermore, inhibition of PLC and MEK or downregulation of arrestin3 leads to a substantial decrease in phosphoBAD levels (Fig. 5D & 5E; Figure S4) supporting the involvement of the arrestin-pERK pathway in mediating BAD phosphorylation in N2A^CB1R^DOR cells (Fig. 6 D & E). The activation of this pathway, which has been implicated in blockade of apoptosis [46], suggests a role for heteromer-directed signaling in cannabinoid-mediated cell survival and differentiation.

### Cell survival is enhanced and apoptosis is decreased in cells co-expressing CB_1_R and DOR

We directly examined Hu210-mediated cell survival in N2A^CB1R^DOR cells, and compared it to N2A^CB1R^ cells. We found a ∼2–4-fold increase in cell survival of N2A^CB1R^DOR compared to N2A^CB1R^ cells (Fig. 6A). We also found that the enhanced survival of N2A^CB1R^DOR cells was due to decreased apoptosis, since the level of caspase-3 activity was reduced by ∼2-fold in N2A^CB1R^DOR compared to N2A^CB1R^ cells (Fig. 6B). Next we tested the involvement of CB_1_R-DOR interactions in cell survival of primary cortical neurons from wild-type and compared that to the survival of neurons from DOR−/− mice. We find that treatment with the CB_1_R antagonist, AM251 leads to a substantial and significant decrease in survival of wild-type cortical neurons; this treatment did not affect survival of neurons from DOR −/− mice (Fig. 6C). This effect of the CB_1_R antagonist AM251, largely absent in neurons lacking DOR, supports the idea that activation of CB_1_R-DOR in the context of the heteromer leads to unique signaling involved in neuronal survival. These observations are relevant in light of recent studies reporting a role for cannabinoid signaling in neuronal development [5]. Therefore our results support the notion that DOR provides CB_1_R with a platform for recruiting new signaling molecules, leading to new levels of receptor regulation and fine-tuning of physiologically important signaling.

In this study, we show that the functional properties of CB_1_R are modulated by co-expression and heteromerization with DOR. We show that: (i) CB_1_R and DOR interacting complexes can be immunoprecipitated from cells expressing these receptors; (ii) CB_1_R receptors exhibit cell surface localization only in the presence of DOR; (iii) arrestin3 is recruited in a PLC dependent fashion to CB_1_R-DOR after Hu210 stimulation in cells expressing both receptors; and (iv) arrestin3 recruitment leads to an alternate pathway for pERK activation, which in turn leads to enhanced cell survival. These results support the notion that CB_1_R and DOR form heteromers, facilitating the promotion of distinct signaling pathways. A schematic summarizing this is shown in Fig. 6 D & E. In N2A^CB1R^ cells, Hu210-mediated activation leads to PLC-dependent pERK activation via the classical G protein-PLC pathway, leading to the phosphorylation of ERK substrates STAT3 and p70S6 kinase (substrates “A”). In N2A^CB1R^DOR cells, Hu210 treatment leads to the PLC-dependent phosphorylation of DOR and recruitment of arrestin3 to the CB_1_R-DOR complex; activation of the arrestin3 pathway can lead to ERK activation and phosphorylation of additional ERK substrates p90rsk and BAD (substrates “B”). Enhanced arrestin3 recruitment to the CB_1_R-DOR complex may be associated with increased CB_1_R desensitization resulting in reduced, G protein-dependent-ERK activation via the classical pathway (leading to phosphorylation of substrates “A”).

The results of this study, together with other recent work, suggest a special role for arrestin3 in directing signals from interacting GPCRs [36]. We have previously shown that direct interaction between MOR and DOR leads to distinct signaling through recruitment of arrestin3 and promotion of arrestin-mediated signaling which, in turn, leads to the phosphorylation of distinct pERK substrates and activation of downstream transcription factor activity [36]. These previous findings, along with our current findings suggest that arrestin3 may play a pivotal role in orchestrating fine tune signaling regulation of GPCR heteromers.

In this study we show that a single cannabinoid agonist (Hu210) is able to elicit distinct signaling responses by binding and activating the same receptor, CB_1_R, based on its association with another GPCR, in this case DOR. We also show that the new pathway activated by CB_1_R in the presence of DOR is involved in the regulation of cell survival, suggesting that GPCR heteromerization contributes to the promotion of context-specific cellular responses, which occur only upon co-expression of the two receptors. Thus, the presence of DOR can influence CB_1_R activity either by direct interactions where DOR could serve as a chaperone helping to target CB_1_R to the cell surface and induce the recruitment of novel signaling molecules to the CB_1_R-DOR heteromer or by indirectly modulating signaling pathways activated by CB_1_R. In the GPCR field, it has recently been established that one GPCR can elicit multiple types of signaling/biological responses when stimulated by different types of ligands; this is termed ‘ligand-directed signaling specificity’ [47], [48]. Thus some agonists have been shown to preferentially activate G protein-mediated pathways while other agonists for the same receptor preferentially activate an arrestin-mediated pathway [49]. In this context, our current findings establish a key role for receptor heteromerization in directing signal specificity in response to a single ligand which we term as ‘heteromer-directed signaling specificity’. We identify the biochemical pathways by which heteromers facilitate distinct signaling cascades and show that activation of a heteromer-specific signaling pathway by a single agonist can induce distinct biological effects.

## Supporting Information

Figure S1 **A–C**, Specificity of rat polyclonal DOR antibody: A, Immunofluorescence with mouse primary cortical cells, 14DIV. Cells from wild type or DOR −/− mice were fixed with 4% PFA in PBS and permeablized with 0.1% Triton, then stained with 1∶500 dilution of rat polyclonal anti-DOR antibody (red) and 1∶1000 dilution of Alexa Fluor 594 goat anti-rat secondary antibodies. Representative of 3 independent experiments shown. **B**, ELISA with cortical membranes from wild type, CB_1_R −/−, and DOR −/− mice. ELISA was carried essentially as described [25], [33], [35] with cortical membranes from wild type, CB_1_R −/−, and DOR −/− mice prepared as described [31]–[33] and 1∶500 dilution of rat polyclonal anti-DOR antibody and 1∶1000 dilution of HRP-conjugated anti-rat secondary antibody. Data represent Mean ± SEM (n = 3 animals/group). **C**, Western blot with cortical membranes from DOR −/− and wild-type mice. Cortical membranes (∼30 µg) from DOR −/− and wild-type mice prepared as described [31]–[33] were subjected to Western blotting using 1∶1000 dilution of rat polyclonal anti-DOR and rabbit polyclonal anti-calnexin (CNX) antibodies and 1∶10,000 dilution of IRDye 680-labeled anti-rat and IRDye 800-labeled anti-rabbit antibodies as described in Methods. (n = 2 animals/group). **D**, CB_1_R and DOR form interacting complexes. Lysates were prepared from N2A cells endogenously expressing CB_1_R (N2A^CB1R^) or stably transfected with either myc tagged DOR (N2A^CB1R^DOR) or Flag tagged DORΔ15 (N2A^CB1R^DORΔ15). (*Left panel*) Lysates (∼30 µg) were subjected to Western blotting with anti-CB_1_R (1∶500) or anti-calnexin (1∶1000) antibodies followed by incubation with IRDye 680-labeled anti-rabbit antibody (1∶10,000) as described in Methods. (*Right panel*) Lysates (100–200 µg) were subjected to immunoprecipitation using either anti-myc or anti-Flag antibodies (1 µg) to pulldown myc tagged DOR or Flag tagged DORΔ15 immunoprecipitates respectively. The immunoprecipitates were resolved on 10% SDS-PAGE and probed for the presence of CB_1_R (1∶500) as described in Methods. Representative of 3 independent experiments shown.
(TIF)

Figure S2 **A**, N2A^CB1R^ or **B**, N2A^CB1R^DOR cells were transfected with a plasma membrane marker (Mb-GFP, green), and the cells were stained with the rabbit polyclonal anti-C-ter CB_1_R antibody (1∶500; magenta) and the mouse monoclonal anti-myc antibody (1∶1000; red). The secondary antibodies used were Alexa 594-coupled goat anti-mouse and Alexa 647-coupled goat anti-rabbit antibodies (1∶1,000). Colocalization of CB_1_R with the plasma membrane marker was examined in horizontal (left panel) and vertical (xzy) (right panel) sections of the cells. The position of xzy section is indicated by a white line. Representative of 3 experiments shown. **C**, Quantification of CB_1_R localized at plasma membrane in N2A^CB1R^ or N2A^CB1R^DOR cells. Using the metamorph software (Molecular Devices), multiple horizontal cross-sections of 8 individual cells per group were used for quantification, and the average of all the values obtained per cell is plotted. Briefly, the percentage of CB_1_R pixels colocalized with Mb-GFP was calculated in each section, and the average for each cell is represented in the graph. ***p<0.001 N2A^CB1R^ vs N2A^CB1R^DOR cells. In colocalization studies we find <5% of CB_1_R colocalize with Mb-GFP in N2A^CB1R^ cells alone, whereas, >40% colocalize with the plasma membrane marker in N2A^CB1R^DOR cells. **D**, Cell surface localization of CB_1_R in N2A^CB1R^ stably expressing DOR or ECE2. Non-permeabilized and permeabilized N2A^CB1R^ stably expressing either DOR or ECE-2 were used to quantify the cell surface and total CB_1_R levels by ELISA as described in Methods. Increase in plasma membrane CB_1_R is seen in N2A^CB1R^DOR cells but not in cells stably expressing a type II transmembrane protein, the metalloprotease endothelin converting enzyme 2 (ECE2), confirming the specificity of DOR in the alteration of CB_1_R localization. Data represents Mean ± SE (n = 3). **E**, Effect of DOR down-regulation on CB_1_R cell surface localization. F11 cells transduced with the DOR shRNA expressing lentivirus, were stained with an anti-N-ter CB_1_R antibody (1∶500) prior to fixation of the cells to label cell surface receptors and visualized using Alexa 594 anti-rabbit antibodies (1∶1000) as described in Methods. Among the cells visualized, the green ones are transduced with the shRNA expressing virus (visualized as green, due to the concurrent expression of nuclear-targeted eGFP). CB_1_R could not be detected at the cell surface of the transduced cells (see arrowheads), whereas CB_1_R was present at the cell surface of untransduced cells (see arrows). **F**, Localization of arrestin3 in N2A^CB1R^ and N2A^CB1R^DOR cells. N2A^CB1R^ and N2A^CB1R^DOR cells transfected with arrestin3-eGFP were stimulated with 100 nM Hu210 for 5 min. Cells were fixed with methanol and incubated with DAPI for nuclear staining (blue). Representative of 3 experiments shown.
(TIF)

Figure S3 **A**, Dose-response of Hu210-mediated ERK phosphorylation in N2A^CB1R^ and N2A^CB1R^DOR cells. Starved N2A^CB1R^ and N2A^CB1R^DOR cells seeded in 24 well-plates were treated with indicated concentrations of Hu210 for 5 minutes. Cell lysates (30 µg protein) were analyzed by Western blotting and probed for the levels of pERK (1∶1000) and ERK (1∶1000) as described in Methods. IRDye 680 anti-rabbit and IRDye 800 anti-mouse were used as secondary antibodies (1∶10,000). Representative blot from 3 independent experiments shown. **B**, Effect of DOR down-regulation on ERK phosphorylation. F11 cells transduced with the DOR shRNA expressing lentivirus were starved for 4–6 h and treated with Hu210 (100 nM) for 5 min. Cell lysates (30 µg protein) were analyzed by Western blotting and probed for the levels of pERK (1∶1000) and ERK (1∶1000). IRDye 680 anti-rabbit and IRDye 800 anti-mouse were used as secondary antibodies (1∶10,000). Representative blot from 3 independent experiments shown. **C**, Effect of PLC inhibitor (edelfosine) on DOR phosphorylation at serine 363 after Hu210 treatment in N2A^CB1R^DOR cells. N2A^CB1R^DOR cells were starved for 4–6 hours, and incubated with vehicle (DMSO) or edelfosine (1 µM) for 30 minutes, then stimulated with 100 nM Hu210 for 5 minutes. Cell lysates (30 µg protein) were subjected to Western blotting and probed for the levels of phospho-DOR Ser 363 (1∶1000) and myc-DOR (1∶1000) as described in Methods. IRDye 680 anti-rabbit and IRDye 800 anti-mouse were used as secondary antibodies (1∶10,000). Data represent Mean ± SEM (n = 3). **D**, Effect of pertussis toxin on Hu210-mediated ERK phosphorylation in N2A^CB1R^ and N2A^CB1R^DOR cells. Starved N2A^CB1R^ and N2A^CB1R^DOR cells pretreated with pertussis toxin as described in Methods were treated with increasing concentrations of Hu210 (0–100 nM) for 5 minutes. Cell lysates (30 µg protein) were analyzed by Western blotting and probed for the levels of pERK (1∶1000) and ERK (1∶1000). IRDye 680 anti-rabbit and IRDye 800 anti-mouse were used as secondary antibodies (1∶10,000). Quantitation of data obtained with 100 nM Hu210 shown. Data represent Mean ± SEM (n = 3 independent experiments).
(TIF)

Figure S4 **A**, Effect of PLC inhibitor (edelfosine) on ERK phosphorylation. N2A^CB1R^ and N2A^CB1R^DOR cells were starved for 4–6 hours, and incubated with vehicle (DMSO) or edelfosine (1 µM) for 30 minutes, then stimulated with 100 nM Hu210 for 5 minutes. Cell lysates (30 µg protein) were subjected to Western blotting and probed for the levels of pERK (1∶1000) and ERK (1∶1000) as described in Methods. IRDye 680 anti-rabbit and IRDye 800 anti-mouse were used as secondary antibodies (1∶10,000). Representative blot from 3 independent experiments shown. **B**, Effect of PLC inhibitor (edelfosine) on STAT3 phosphorylation. N2A^CB1R^ and N2A^CB1R^DOR cells were starved for 4–6 hours, and incubated with vehicle (DMSO) or edelfosine (1 µM) for 30 minutes, then stimulated with 100 nM Hu210 for 5 minutes. Cell lysates (30 µg protein) were subjected to Western blotting and probed for the levels of pSTAT3 (1∶1000) and STAT3 (1∶1000) as described in Methods. IRDye 680 anti-rabbit and IRDye 800 anti-mouse were used as secondary antibodies (1∶10,000). Representative blot from 3 independent experiments shown. **C**, Effect of PLC inhibitor (edelfosine) on BAD phosphorylation. N2A^CB1R^ and N2A^CB1R^DOR cells were starved for 4–6 hours, and incubated with vehicle (DMSO) or edelfosine (1 µM) for 30 minutes, then stimulated with 100 nM Hu210 for 5 minutes. Cell lysates (30 µg protein) were subjected to Western blotting and probed for the levels of pBAD (1∶1000) and BAD (1∶1000) as described in Methods. IRDye 680 anti-rabbit and IRDye 800 anti-mouse were used as secondary antibodies (1∶10,000). Representative blot from 3 independent experiments shown.
(TIF)
