# Supplementary figures and images for: Receptor Heteromerization Expands the Repertoire of Cannabinoid Signaling in Rodent Neurons
Source: PLoS One. 2012 Jan 3;7(1):e29239. doi: 10.1371/journal.pone.0029239 (PMC3250422; doi:10.1371/journal.pone.0029239)

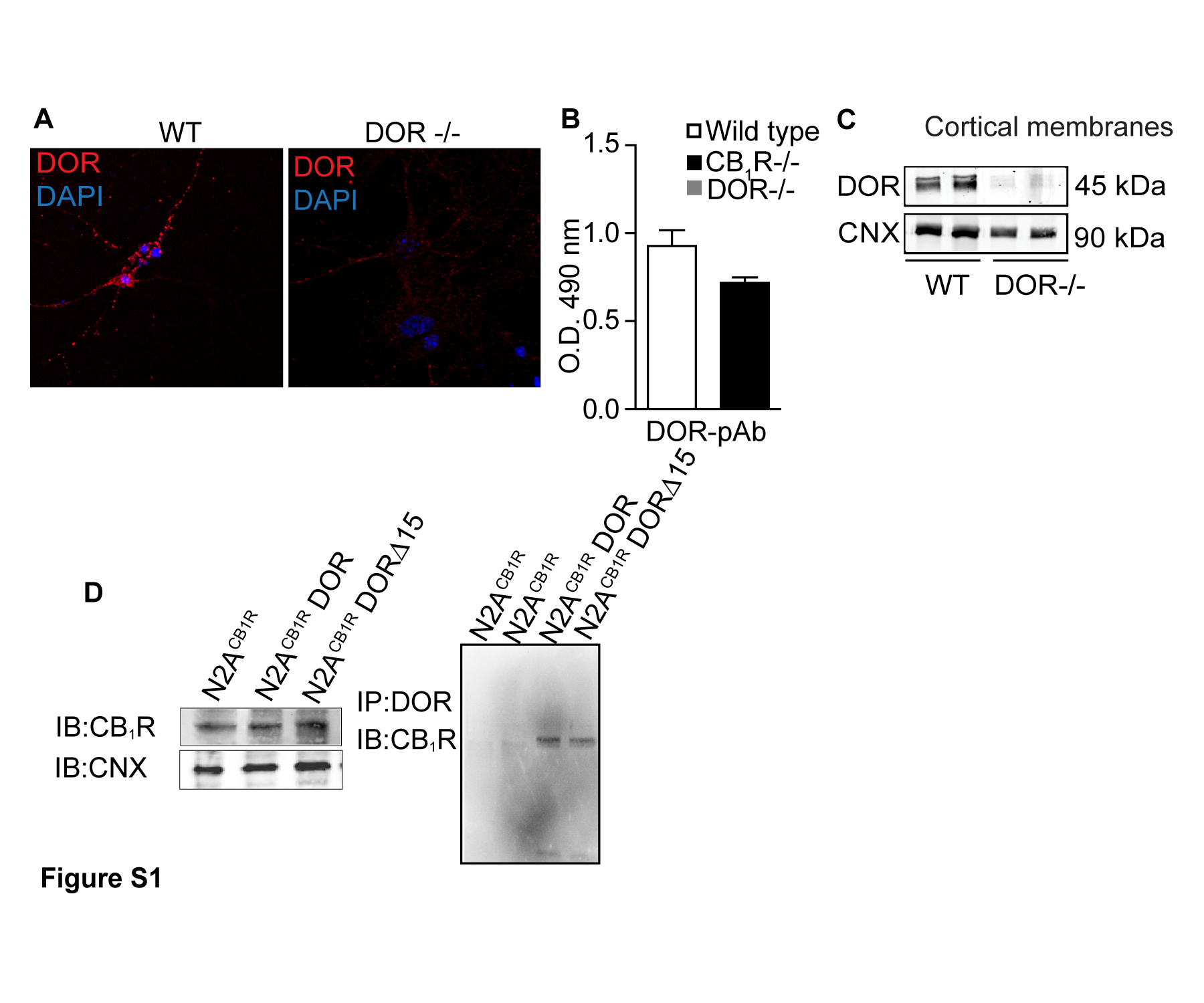

Supplement: Figure S1 — A–C, Specificity of rat polyclonal DOR antibody: A, Immunofluorescence with mouse primary cortical cells, 14DIV. Cells from wild type or DOR −/− mice were fixed with 4% PFA in PBS and permeablized with 0.1% Triton, then stained with 1∶500 dilution of rat polyclonal anti-DOR antibody (red) and 1∶1000 dilution of Alexa Fluor 594 goat anti-rat secondary antibodies. Representative of 3 independent experiments shown. B, ELISA with cortical membranes from wild type, CB1R −/−, and DOR −/− mice. ELISA was carried essentially as described [25], [33], [35] with cortical membranes from wild type, CB1R −/−, and DOR −/− mice prepared as described [31]–[33] and 1∶500 dilution of rat polyclonal anti-DOR antibody and 1∶1000 dilution of HRP-conjugated anti-rat secondary antibody. Data represent Mean ± SEM (n = 3 animals/group). C, Western blot with cortical membranes from DOR −/− and wild-type mice. Cortical membranes (∼30 µg) from DOR −/− and wild-type mice prepared as described [31]–[33] were subjected to Western blotting using 1∶1000 dilution of rat polyclonal anti-DOR and rabbit polyclonal anti-calnexin (CNX) antibodies and 1∶10,000 dilution of IRDye 680-labeled anti-rat and IRDye 800-labeled anti-rabbit antibodies as described in Methods. (n = 2 animals/group). D, CB1R and DOR form interacting complexes. Lysates were prepared from N2A cells endogenously expressing CB1R (N2ACB1R) or stably transfected with either myc tagged DOR (N2ACB1RDOR) or Flag tagged DORΔ15 (N2ACB1RDORΔ15). (Left panel) Lysates (∼30 µg) were subjected to Western blotting with anti-CB1R (1∶500) or anti-calnexin (1∶1000) antibodies followed by incubation with IRDye 680-labeled anti-rabbit antibody (1∶10,000) as described in Methods. (Right panel) Lysates (100–200 µg) were subjected to immunoprecipitation using either anti-myc or anti-Flag antibodies (1 µg) to pulldown myc tagged DOR or Flag tagged DORΔ15 immunoprecipitates respectively. The immunoprecipitates were resolved on 10% SDS-PAGE and probed for the p [file pone.0029239.s001.tif]

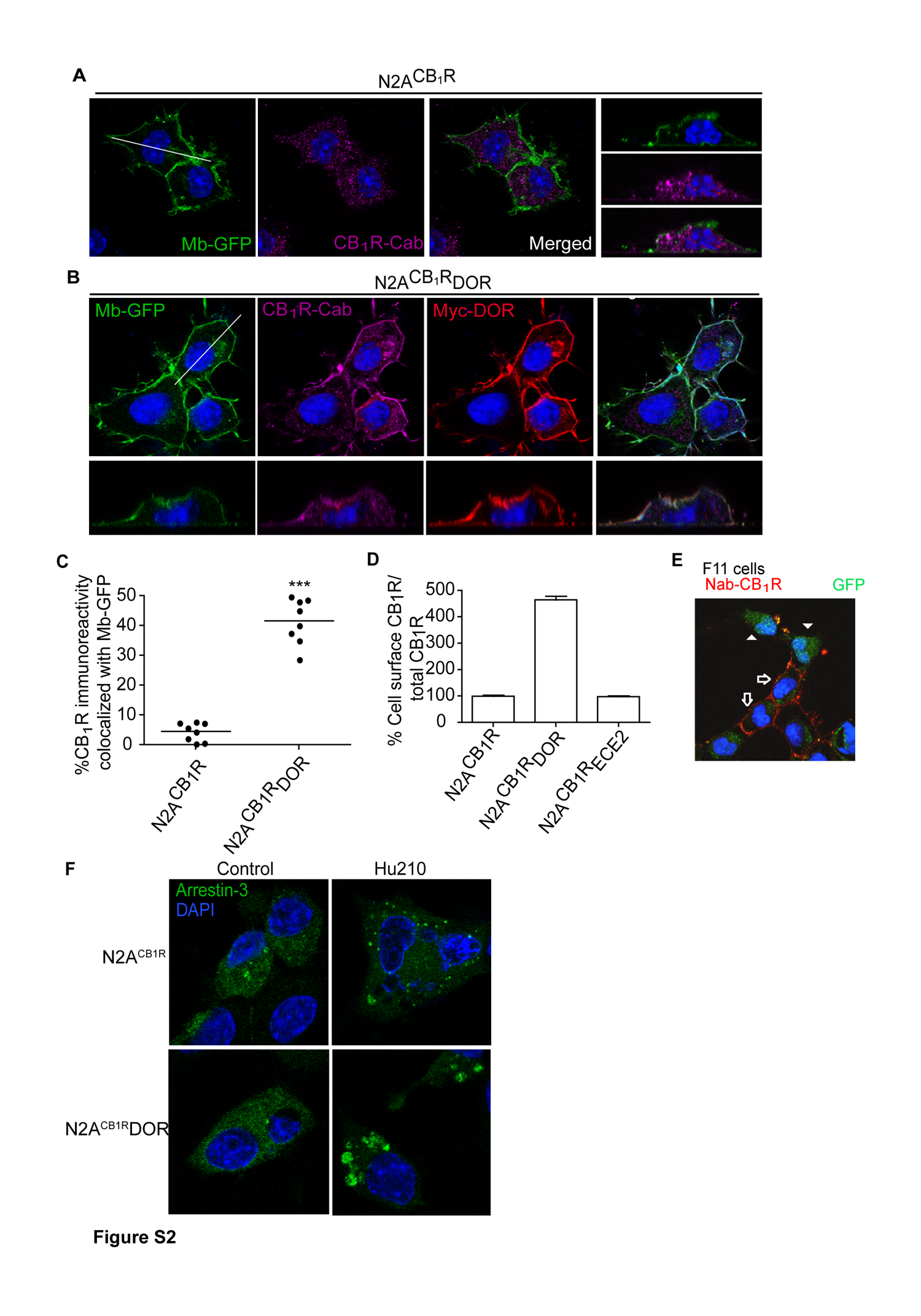

Supplement: Figure S2 — A, N2ACB1R or B, N2ACB1RDOR cells were transfected with a plasma membrane marker (Mb-GFP, green), and the cells were stained with the rabbit polyclonal anti-C-ter CB1R antibody (1∶500; magenta) and the mouse monoclonal anti-myc antibody (1∶1000; red). The secondary antibodies used were Alexa 594-coupled goat anti-mouse and Alexa 647-coupled goat anti-rabbit antibodies (1∶1,000). Colocalization of CB1R with the plasma membrane marker was examined in horizontal (left panel) and vertical (xzy) (right panel) sections of the cells. The position of xzy section is indicated by a white line. Representative of 3 experiments shown. C, Quantification of CB1R localized at plasma membrane in N2ACB1R or N2ACB1RDOR cells. Using the metamorph software (Molecular Devices), multiple horizontal cross-sections of 8 individual cells per group were used for quantification, and the average of all the values obtained per cell is plotted. Briefly, the percentage of CB1R pixels colocalized with Mb-GFP was calculated in each section, and the average for each cell is represented in the graph. ***p<0.001 N2ACB1R vs N2ACB1RDOR cells. In colocalization studies we find <5% of CB1R colocalize with Mb-GFP in N2ACB1R cells alone, whereas, >40% colocalize with the plasma membrane marker in N2ACB1RDOR cells. D, Cell surface localization of CB1R in N2ACB1R stably expressing DOR or ECE2. Non-permeabilized and permeabilized N2ACB1R stably expressing either DOR or ECE-2 were used to quantify the cell surface and total CB1R levels by ELISA as described in Methods. Increase in plasma membrane CB1R is seen in N2ACB1RDOR cells but not in cells stably expressing a type II transmembrane protein, the metalloprotease endothelin converting enzyme 2 (ECE2), confirming the specificity of DOR in the alteration of CB1R localization. Data represents Mean ± SE (n = 3). E, Effect of DOR down-regulation on CB1R cell surface localization. F11 cells transduced with the DOR shRNA expressing lentivirus, were stained with an an [file pone.0029239.s002.tif]

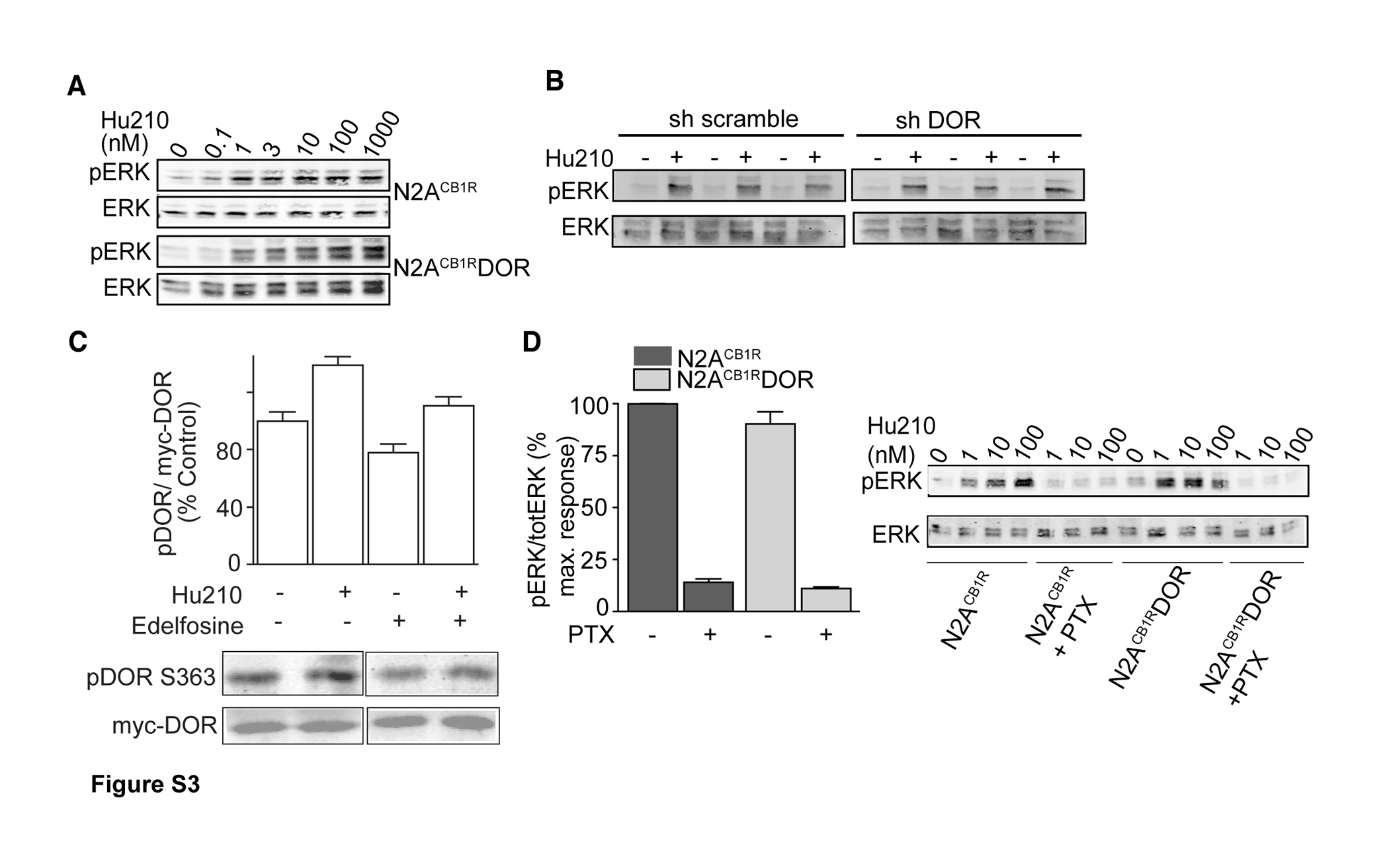

Supplement: Figure S3 — A, Dose-response of Hu210-mediated ERK phosphorylation in N2ACB1R and N2ACB1RDOR cells. Starved N2ACB1R and N2ACB1RDOR cells seeded in 24 well-plates were treated with indicated concentrations of Hu210 for 5 minutes. Cell lysates (30 µg protein) were analyzed by Western blotting and probed for the levels of pERK (1∶1000) and ERK (1∶1000) as described in Methods. IRDye 680 anti-rabbit and IRDye 800 anti-mouse were used as secondary antibodies (1∶10,000). Representative blot from 3 independent experiments shown. B, Effect of DOR down-regulation on ERK phosphorylation. F11 cells transduced with the DOR shRNA expressing lentivirus were starved for 4–6 h and treated with Hu210 (100 nM) for 5 min. Cell lysates (30 µg protein) were analyzed by Western blotting and probed for the levels of pERK (1∶1000) and ERK (1∶1000). IRDye 680 anti-rabbit and IRDye 800 anti-mouse were used as secondary antibodies (1∶10,000). Representative blot from 3 independent experiments shown. C, Effect of PLC inhibitor (edelfosine) on DOR phosphorylation at serine 363 after Hu210 treatment in N2ACB1RDOR cells. N2ACB1RDOR cells were starved for 4–6 hours, and incubated with vehicle (DMSO) or edelfosine (1 µM) for 30 minutes, then stimulated with 100 nM Hu210 for 5 minutes. Cell lysates (30 µg protein) were subjected to Western blotting and probed for the levels of phospho-DOR Ser 363 (1∶1000) and myc-DOR (1∶1000) as described in Methods. IRDye 680 anti-rabbit and IRDye 800 anti-mouse were used as secondary antibodies (1∶10,000). Data represent Mean ± SEM (n = 3). D, Effect of pertussis toxin on Hu210-mediated ERK phosphorylation in N2ACB1R and N2ACB1RDOR cells. Starved N2ACB1R and N2ACB1RDOR cells pretreated with pertussis toxin as described in Methods were treated with increasing concentrations of Hu210 (0–100 nM) for 5 minutes. Cell lysates (30 µg protein) were analyzed by Western blotting and probed for the levels of pERK (1∶1000) and ERK (1∶1000). IRDye 680 anti-rabbit and IRDye 800 anti-mouse [file pone.0029239.s003.tif]

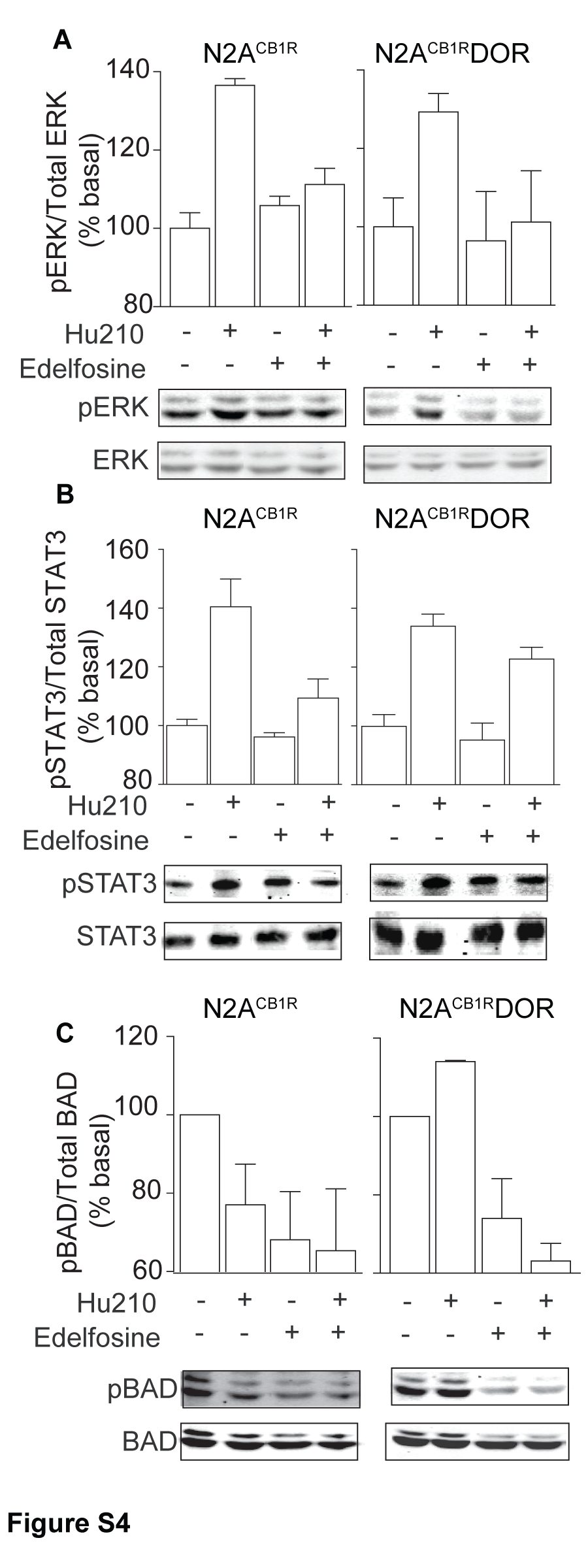

Supplement: Figure S4 — A, Effect of PLC inhibitor (edelfosine) on ERK phosphorylation. N2ACB1R and N2ACB1RDOR cells were starved for 4–6 hours, and incubated with vehicle (DMSO) or edelfosine (1 µM) for 30 minutes, then stimulated with 100 nM Hu210 for 5 minutes. Cell lysates (30 µg protein) were subjected to Western blotting and probed for the levels of pERK (1∶1000) and ERK (1∶1000) as described in Methods. IRDye 680 anti-rabbit and IRDye 800 anti-mouse were used as secondary antibodies (1∶10,000). Representative blot from 3 independent experiments shown. B, Effect of PLC inhibitor (edelfosine) on STAT3 phosphorylation. N2ACB1R and N2ACB1RDOR cells were starved for 4–6 hours, and incubated with vehicle (DMSO) or edelfosine (1 µM) for 30 minutes, then stimulated with 100 nM Hu210 for 5 minutes. Cell lysates (30 µg protein) were subjected to Western blotting and probed for the levels of pSTAT3 (1∶1000) and STAT3 (1∶1000) as described in Methods. IRDye 680 anti-rabbit and IRDye 800 anti-mouse were used as secondary antibodies (1∶10,000). Representative blot from 3 independent experiments shown. C, Effect of PLC inhibitor (edelfosine) on BAD phosphorylation. N2ACB1R and N2ACB1RDOR cells were starved for 4–6 hours, and incubated with vehicle (DMSO) or edelfosine (1 µM) for 30 minutes, then stimulated with 100 nM Hu210 for 5 minutes. Cell lysates (30 µg protein) were subjected to Western blotting and probed for the levels of pBAD (1∶1000) and BAD (1∶1000) as described in Methods. IRDye 680 anti-rabbit and IRDye 800 anti-mouse were used as secondary antibodies (1∶10,000). Representative blot from 3 independent experiments shown. (TIF) [file pone.0029239.s004.tif]
